# Sharing tasks or sharing actions? Evidence from the joint Simon task

**DOI:** 10.1007/s00426-016-0821-y

**Published:** 2016-11-08

**Authors:** Motonori Yamaguchi, Helen J. Wall, Bernhard Hommel

**Affiliations:** 1 Department of Psychology, Edge Hill University, Ormskirk, UK; 2 Institute of Psychology, Leiden University, Leiden, The Netherlands

## Abstract

In a joint Simon task, a pair of co-acting individuals divide labors of performing a choice-reaction task in such a way that each actor responds to one type of stimuli and ignores the other type that is assigned to the co-actor. It has been suggested that the actors share the mental representation of the joint task and perform the co-actor’s trials as if they were their own. However, it remains unclear exactly which aspects of co-actor’s task-set the actors share in the joint Simon task. The present study addressed this issue by manipulating the proportions of compatible and incompatible trials for one actor (inducer actor) and observing its influences on the performance of the other actor (diagnostic actor) for whom there were always an equal proportion of compatible and incompatible trials. The design of the present study disentangled the effect of trial proportion from the confounding effect of compatibility on the preceding trial. The results showed that the trial proportions for the inducer actor had strong influences on the inducer actor’s own performance, but it had little influence on the diagnostic actor’s performance. Thus, the diagnostic actor did not represent aspects of the inducer actor’s task-set beyond stimuli and responses of the inducer actor. We propose a new account of the effect of preceding compatibility on the joint Simon effect.

## Introduction

One of the key skills to succeed in highly competitive environments is to collaborate with others to achieve a common goal (Bedwell et al., 2012). True collaboration requires more than merely sharing a goal, as the contributions of one co-actor often need to be coordinated with the contributions of other co-actors in terms of content, space, and time. Hence, working on the same goal requires co-actors to take into account contributions from others to some degree. This raises the question of which aspects of the co-actor’s contributions people represent in a collaborative task setting. Successful collaboration would require monitoring the actions that a co-actor performs, and the stimuli to which these actions are performed (Yamaguchi, Wall, & Hommel, 2016). Importantly for the present context, it has been suggested that people automatically *co*-*represent* the co-actors’ task parameters (Sebanz, Knoblich, & Prinz, 2003; Knoblich, Butterfill, & Sebanz, 2011), in a way that “actions at another person’s command are represented just as if they were at one’s own command” (Knoblich & Sebanz, 2006, p. 101). Co-representation of this kind is said to take place when an individual shares another individual’s mental representation (Sebanz, Knoblich, & Prinz, 2005). Hence, we understand the notion of task co-representation to mean that co-acting individuals integrate into their task representations their co-actor’s *task*-*set*, which involves task parameters such as stimuli, responses, and their relations, that their co-actor face. To date, it remains unclear as to what aspects of co-actor’s task parameters people actually represent in order to perform a joint task.

The paradigm used most often to investigate the degree to which people represent aspects of co-actors and their activities is the joint Simon task (Sebanz et al., 2003). In a standard, individual Simon task, participants are to press a left or right key in response to non-spatial features of a stimulus that appears randomly to the left or right of some reference point, such as a fixation point at the center of a screen. Even though stimulus location is irrelevant to selecting the correct response, responses are faster and more accurate if the stimulus location coincides with the response location (compatible trial) than if it does not (incompatible trial), which is known as the Simon effect (Lu & Proctor, 1995; Yamaguchi & Proctor, 2012). In the *joint Simon task*, the two responses are divided between two co-acting participants, in such a way that one participant responds to one type of stimuli (e.g., red stimuli) while the other responds to the other stimulus type (e.g., green stimuli). This manipulation renders the task essentially a go/nogo task, which commonly does not yield a significant Simon effect in the absence of a co-actor (Hommel, 1996). In the presence of an active co-actor, however, reliable Simon effects are observed (Sebanz et al., 2003).

Given that the Simon effect is attributed to response-selection processes (Hommel, 2011; Lu & Proctor, 1995), the joint Simon effect indicates that, when selecting their own responses, participants take the active contribution (i.e., response) of their co-actor into consideration. Further evidence suggests that participants also consider the stimuli that a co-actor is responding to. In the standard, individual Simon task, the Simon effect is known to be more pronounced after a compatible trial than after an incompatible trial (Stürmer, Leuthold, Soetens, Schröter, & Sommer, 2002), whereby the Simon effect after an incompatible trial is often non-significant or even reversed to favor an incompatible response (Proctor, Yamaguchi, Dutt, & Gonzalez, 2013). Such sequential modulations of the Simon effect are thought to represent reactive adjustments of control settings (an increase of attention weights on relevant information after experiencing conflict in an incompatible trial; Botvinick, Cohen, & Carter, 2004), priming of an earlier stimulus-episode (Hommel, Proctor, & Vu, 2004), or both. Interestingly, comparable sequential modulations are obtained in the joint Simon task even on trials that follow a response of the co-actor (Liepelt, Wenke, Fischer, & Prinz, 2011; Liepelt, Wenke, & Fischer, 2013), which implies that participants represent not only the co-actor’s response but also the stimulus that triggers the response. While it seems well-established that actors consider their co-actor’s stimuli and responses when performing a joint Simon task (for a review, see Dolk et al., 2014), it remains to be seen whether they spontaneously represent the entire task-set of the co-actor that includes representations of the stimulus–response relationship as well as contextual factors such as the frequencies of particular trial events.

Therefore, the main aim of the present study was to test to what degree participants in the joint Simon task represent aspects of their co-actor’s task-set. We did so by manipulating the proportions of compatible and incompatible trials that are known to affect the Simon effect in the standard version of the task (Hommel, 1994). In the standard Simon task, the probabilities of compatible and incompatible trials are equated to rule out the possibility that participants predict responses from stimulus location. When the overall proportion of compatible trials is greater than 50% (*mostly compatible block*), stimulus location allows participants to predict that the correct response is spatially compatible in most cases, which increases the Simon effect. In contrast, the Simon effect decreases when the overall proportion of compatible trials is smaller than 50% (*mostly incompatible block*). These observations are taken to reflect proactive control adjustments that increase the impact of stimulus location on response selection, and the proportion-of-compatibility effect reflects the actor’s contextualized task-sets that are adjusted according to the task context. Hence, the manipulation of trial proportions provides an ideal testbed to examine the influence of a shared task-set on joint performance.

To test what aspects of the co-actor’s task parameters actors represent in performing the joint Simon task, the present study manipulated the trial proportions for one actor (*inducer actor*), by making either compatible or incompatible trials more probable than the other in a given experimental block, while keeping the trial proportions for the other actor (*diagnostic actor*) equal across all blocks. The Simon effect for the inducer actor should increase when compatible trials are more probable (in the mostly compatible block), but the effect should decrease when incompatible trials are more probable (in the mostly incompatible block), reflecting his or her own contextualized task-set. This result should not depend on whether the inducer actor shares the diagnostic actor’s task-set.

Of more importance is the effect of trial proportions on the diagnostic actor’s performance. If the diagnostic actor shares the inducer actor’s task-set, the diagnostic actor should consider the proportions of compatible and incompatible trials for the inducer actor as if they were his or her own trials. Thus, the diagnostic actor’s Simon effect should increase in the mostly compatible block, and decrease in the mostly incompatible block, as much as the inducer actor’s Simon effect does in that block. Previous studies of individual tasks have shown that the effect of trial proportions do transfer across two different tasks that are intermixed randomly (Wühr, Duthoo, & Notebaert, 2015). We expected that the effect of trial proportions would also transfer across two actors as long as they monitored the trial proportions for their co-actors. If the diagnostic actor does not share the inducer actor’s task-set, however, the diagnostic actor should not consider the proportions of compatible and incompatible trials for the inducer actor. Thus, the diagnostic actor’s Simon effect should not be influenced by the proportion-of-compatibility manipulation (as this concerns the inducer actor’s trials only) and should be of the same size in all blocks.

However, caution needs to be exercised because the effect of trial proportion is usually confounded by the effect of compatibility on the previous trial. Any imbalance in the proportions of compatible and incompatible trials affect the probability of trial transitions because, for example, having a high proportion of compatible trials renders it more likely for a trial to follow a compatible rather than an incompatible trial. Previous studies have shown that the Simon effect depended on whether the preceding trial was compatible or incompatible in both the joint task and the individual task (Liepelt et al., 2011), indicating that these outcomes are not due to sharing a task between co-actors. Thus, although the proportion-of-compatibility manipulation might not affect the diagnostic actor’s performance directly, it could do so indirectly by altering the proportions of trial transitions. If so, the diagnostic actor’s Simon effect may increase in the mostly compatible block and decrease in the mostly incompatible block, not because the diagnostic actor shares the inducer actor’s task-set, but because there are more trial transitions that the proportion-of-compatibility manipulation favors. Specifically, there would be more transitions from compatible to either compatible or incompatible trials (which are known to increase the Simon effect) in the mostly compatible block, and more transitions from incompatible to either compatible or incompatible trials (which are known to reduce the Simon effect) in the mostly incompatible block.

To elucidate this problem, we examined the proportion-of-compatibility effect separately for trials that followed the inducer actor’s trials and for trials that followed the diagnostic actor’s own trials. When the preceding trial was the inducer actor’s (who had biased proportions of compatible and incompatible trials), the current trials were more likely to follow trials that occurred more frequently; thus, the effect of trial proportion was confounded by the effect of compatibility on the preceding trial. Therefore, it was expected that the diagnostic actor’s Simon effect should increase if the inducer actor is facing a mostly compatible block and decrease if the inducer actor is facing a mostly incompatible block. Yet, these results would not necessarily be because the diagnostic actor is sharing the inducer actor’s task-set, but because of the proportions of trial transitions that the proportion-of-compatibility manipulation favors, as described above.

When the preceding trial was the diagnostic actor’s (who had an equal proportion of compatible and incompatible trials), however, trials were equally likely to follow compatible and incompatible trials; thus, there were no bias in the trial transitions, so the effect of trial proportion was de-confounded from the effect of previous compatibility. Therefore, the influences of the proportion-of-compatibility effect on the diagnostic actor’s Simon effect after the diagnostic actor’s own trials would indicate whether the diagnostic actor shares the inducer actor’s task-set. It was expected that, if the diagnostic actor shares the inducer actor’s task-set, the Simon effect should depend on whether the inducer faces a mostly compatible or incompatible block; if the diagnostic actor does not share the inducer actor’s task-set, the Simon effect should not depend on whether the inducer faces a mostly compatible or incompatible block.

The present study included two sessions. The first session tested the individual and joint Simon tasks, without any proportion-of-compatibility manipulation. This session aimed at replicating main findings in the previous studies, namely, that (1) the Simon effect is obtained only in the joint task but not in the individual task (Sebanz et al., 2003), and that (2) the Simon effect is larger on trials that follow compatible trials than on trials that follow incompatible trials in both the joint and individual tasks (Liepelt et al., 2011). The second session tested the joint task in which the proportions of compatible and incompatible trials were manipulated for the inducer actor across separate blocks while the proportions were kept equal for the diagnostic actor in all blocks. This session aimed at observing the impact of the proportion-of-compatibility manipulation on the diagnostic actor’s Simon effect after the diagnostic actor’s own trials, which would reveal whether the diagnostic actor shares the inducer actor’s task-set.

## Method

### Participants

There were two sessions in the present study. One hundred undergraduate students at Edge Hill University participated in the first session (65 female, 17 male, 18 undeclared; age range 18–43, *M* = 17.70, SD = 4.19), and 80 students came back 4 months later for the second session (61 female, 16 male, 3 undeclared; age range 18–44, *M* = 19.78, SD = 4.08). The experiments were conducted as part of seminar activities in an introductory psychology module. Participants received course credits toward their module or were paid £3 for participation in each session. All reported having normal or corrected-to-normal visual acuity and normal color vision. They were naive as to the purposes of the experiment.

### Apparatus and stimuli

The apparatus consisted of a personal computer and a 19-in. flat-screen monitor with a standard QWERTY keyboard. Stimuli were green and red filled circles (4.5 cm in diameter) presented on the computer monitor at a distance of 10 cm to the left or right of the screen center. Responses were made by pressing a left (“z”) or right (“/”) key to the color of stimuli. The same apparatus and stimuli were used in the two sessions.

### Procedure

In both sessions, all participants were run in the same afternoon. The experimenters paired participants randomly and divided into four groups of similar sizes in each session. Two groups were run in parallel in different computer labs with the same room layouts, which consisted of four rows of six identical computers each. Two adjacent computers were 160 cm apart from each other. Each pair used one computer, and pairs were seated at every other computer. The participant seated on the left pressed the left key, and the participant seated on the right pressed the right key. For half of the pairs, the left and right keys were assigned to red and green circles, respectively; for the other half, the mappings were reversed. Participants were asked not to talk with their partner while performing the experimental task. The task started with on-screen instructions, which asked participants to respond to one type of stimuli as quickly as they could and refrain from responding to the other type. A session took about 30 min.

*Session 1* Each pair performed four blocks. Two blocks consisted of the joint task in which two participants responded to their assigned stimuli. Two remaining blocks consisted of the individual task, one block for each participant, whereby only one participant responded to the assigned stimuli and ignored the other stimuli; the other participant only watched their partner’s trials quietly (Sebanz et al., 2003). Half the pairs started the experiment with the joint task, and the other half started with the individual task. Each block consisted of 80 test trials. There were 12 practice trials before the joint task block and before each of the individual task blocks.

In the joint task block, each trial started with a fixation cross at the screen center for 750 ms, followed by the stimulus that remained on screen for 1000 ms or until a response. The message “Error!” appeared for an error response, “Faster!” for no response, and a blank display for a correct response, for 500 ms. The feedback display was followed by the fixation cross for the next trial. The locations of stimulus and response were compatible in half the trials and incompatible for the other half. Response time (RT) was the interval between stimulus onset and a response. In the individual task block, the only differences were the feedback messages, which were “Respond!” for no response on a go trial and “Do not respond!” for a response on a nogo trial. Go and nogo trials occurred equally frequently with the equal number of compatible and incompatible trials each.

*Session 2* There were three blocks of 160 trials each under the joint task. One of the paired participants was the *inducer actor* for whom the proportions of compatible and incompatible trials were varied across blocks: In the first block, the proportions of compatible and incompatible trials were equal (*equal proportion block*). In one of the following two blocks, 90% of the trials were compatible (*mostly compatible block*); in the other block, 10% of the trials were compatible (*mostly incompatible block*). The order of the two biased conditions was counterbalanced across pairs. The other participant was designated as the *diagnostic actor* for whom the proportions were kept equal for all three blocks. Combining all trials for the two actors, 70% of the trials were compatible for the mostly compatible condition, 70% of the trials were incompatible for the mostly incompatible condition. Participants were not informed of these proportions (Hommel, 1994; Proctor et al., 2013). For half of the pairs, the diagnostic actor sat on the left side; for the other half, the inducer actor sat on the left side.

## Results

Mean RT for correct responses was computed for each participant (the overall error rates were 0.94% in Session 1 and 1.28% in Session 2). For the purpose of testing the effect of trial sequence, the first trial in each block was excluded in the analysis. Also, trials for which no response was made within the 1000-ms response window or RT was less than 200 ms were discarded (0.21 and 0.46% of all trials in Sessions 1 and 2, respectively).

**Fig. 1:**
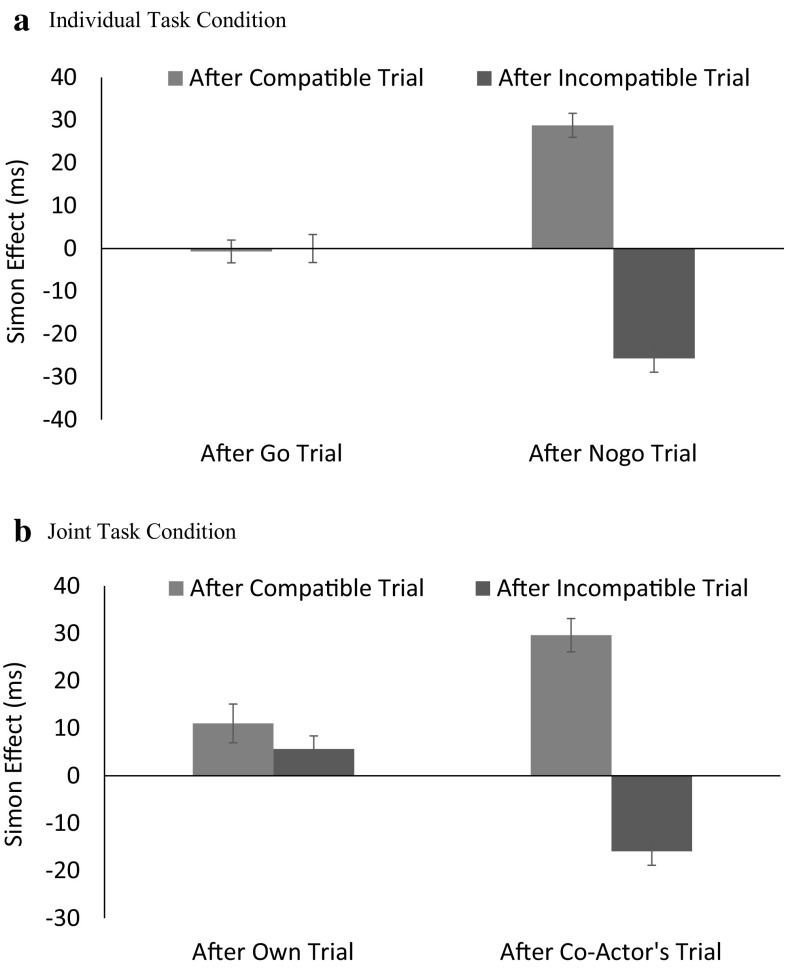
The Simon effect for the individual (**a**) and joint (**b**) conditions in Session 1. *Error bars* represent one standard error of the means

*Session 1* The purpose of Session 1 was to test whether the standard pattern of a significant Simon effect in the joint task but not in the individual task (Sebanz et al., 2003), and whether the previously obtained effect of compatibility on the preceding trial (Liepelt et al., 2011) could be replicated in our experimental setup. To test our predictions, RT was submitted to a 2 (Task: individual vs. joint) × 2 (Preceding Trial: go vs. nogo) × 2 (Preceding Compatibility: compatible vs. incompatible) × 2 (Current Compatibility: compatible vs. incompatible) repeated-measures ANOVA (see Table 1). The following discussions are based on significant effects involving the Simon effect and comparisons between the two task conditions. RTs are summarized in Table 2. The Simon effects are shown in Fig. 1.

**Table 1:** ANOVA results in Session 1

Factor	*df*	MSE	*F*	*p*	*η*_p_^2^
Task (T)	**1, 99**	**2127.91**	**16.75**	**<.001**	**.145**
Previous Actor (PA)	**1, 99**	**1714.05**	**2.78**	**.012**	**.061**
Previous compatibility (PC)	1, 99	290.21	1.00	.319	.010
Current Compatibility (CC)	**1, 99**	**716.52**	**9.57**	**.003**	**.880**
T × PA	1, 99	771.07	1.05	.308	.010
T × PC	1, 99	419.93	3.26	.074	.032
PA × PC	1, 99	415.37	3.25	.075	.032
T × PA × PC	**1, 99**	**418.37**	**7.09**	**.009**	**.067**
T × CC	**1, 99**	**455.95**	**10.53**	**.002**	**.096**
PA × CC	1, 99	571.89	<1	.952	<.001
T × PA × CC	1, 99	392.54	<1	.416	.007
PC × CC	**1, 99**	**550.71**	**123.81**	**<.001**	**.556**
T × PC × CC	1, 99	359.26	<1	.737	.001
PA × PC × CC	**1, 99**	**499.87**	**114.26**	**<.001**	**.536**
T × PA × PC × CC	1, 99	520.83	2.81	.097	.028

Bold indicates significant effect at alpha = .05

**Table 2:** Response times (in millisecond) in Session 1 (values in parentheses are standard errors of the means)

	Compatible	Incompatible
Individual task		
After go trial		
After compatible	343 (4.52)	342 (4.60)
After incompatible	344 (4.95)	344 (5.06)
After nogo trial		
After compatible	336 (3.86)	364 (4.13)
After incompatible	363 (4.01)	337 (4.31)
Joint task		
After own trial		
After compatible	333 (4.47)	344 (4.77)
After incompatible	329 (4.03)	334 (4.01)
After co-actor’s trial		
After compatible	323 (3.72)	353 (4.34)
After incompatible	348 (3.63)	332 (4.26)

There was a small but significant overall Simon effect; RTs were 340 and 344 ms for compatible and incompatible trials, respectively. The Simon effect depended on task. Contrast tests showed that the effect was significant only in the joint task (*M* = 8 ms; *p* < .001), but not in the individual task (*M* = 1 ms; *p* = .656). The Simon effect was larger after a compatible trial (17 ms) than after an incompatible trial (−9 ms). This effect of previous compatibility was apparent on trials after a nogo trial (Simon effects = 29 and −21 ms after compatible and incompatible trials, respectively) but not on the trials after a go trial (Simon effects = 5 vs. 3 ms). Although not relevant to the purpose of the experiment, RT was faster when the preceding trial was the same type (go or nogo) as the current trial than when it was a different type in the individual task (MD = 7 ms), but this advantage was apparent when the preceding trial was incompatible (MD = 8 ms) but not when it was compatible in the joint task (MD = –1 ms). No other effects involving Task were significant. These outcomes agree with those obtained in previous studies (Liepelt et al., 2011; Sebanz et al., 2003).

Of particular interest for the assessment of the findings from Session 2 is the comparison between the general patterns obtained for the joint and individual task conditions: pronounced effects of previous compatibility of almost identical sizes after the co-actor’s trials and nogo trials but little or no effect after having actively performed in the previous trial. In other words, the effect of previous compatibility is more or less restricted to trials following nogo trials in which the participant did not respond. Moreover, it is interesting to note that this pattern resulted from particularly elevated RTs in incompatible trials that followed compatible trials and incompatible trials that followed compatible trials (see Table 2). We will discuss implications of these findings in the Discussion.

**Fig. 2:**
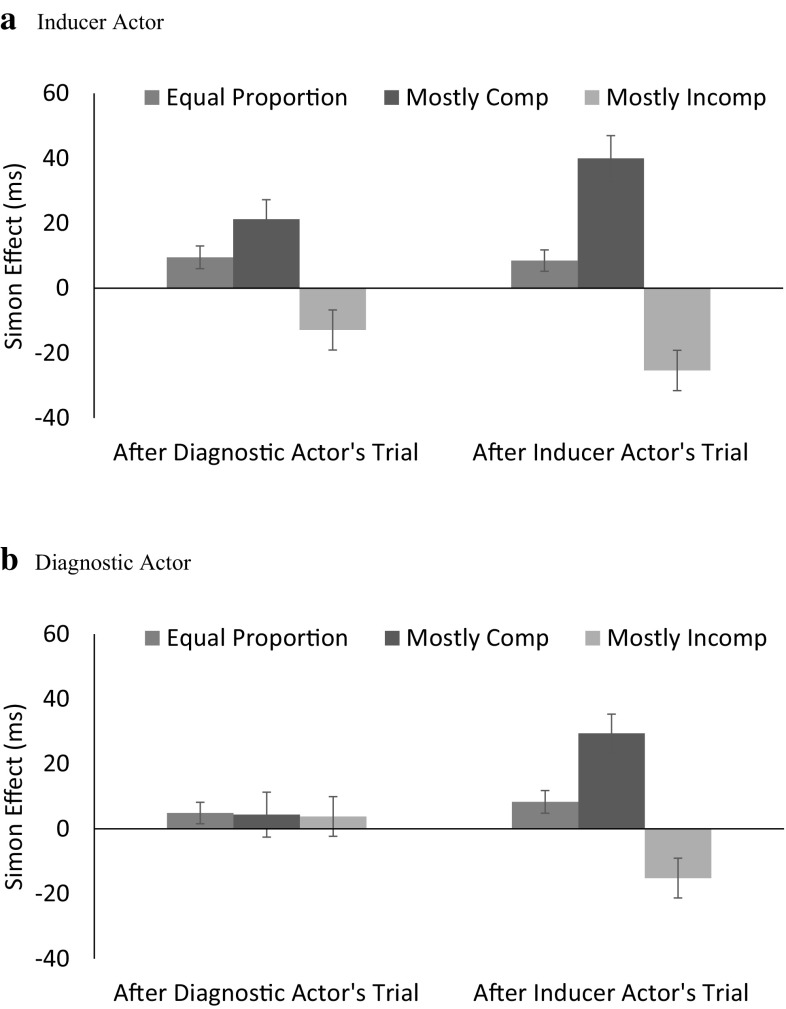
The Simon effect for inducer (**a**) and diagnostic (**b**) actors after the diagnostic actor’s trials and inducer actor’s trials in Session 2. *Error bars* represent one standard error of the means

*Session 2* The main purpose of Session 2 was to compare the effect of trial proportion with respect to the actor who performed the preceding trial. One inducer actor was excluded due to an empty cell in one of the biased proportion conditions, leaving 79 valid participants. RT was submitted to a 3 (Proportion: mostly compatible vs. equal proportion vs. mostly incompatible) × 2 (Previous Actor: same vs. different) × 2 (Current Compatibility: compatible vs. incompatible) × 2 (Current Actor: diagnostic vs. inducer) ANOVA^1^ (see Table 3). RTs are summarized in Table 4, and the Simon effects are shown in Fig. 2.

**Table 3:** ANOVA results in Session 2

Factor	*df*	MSE	*F*	*p*	*η*_p_^2^
Between-subject					
Current Actor (CA)	1, 77	9383.64	3.78	.056	.047
Within-subject					
Proportion (Pr)	**2, 154**	**1032.74**	**19.76**	**<.001**	**.204**
Pr × CA	2, 154	1032.74	1.57	.214	.020
Previous Actor (PA)	**1, 77**	**1418.45**	**11.67**	**.001**	**.132**
PA × CA	1, 77	1418.45	<1	.514	.006
Current compatibility (CC)	**1, 77**	**474.36**	**20.26**	**<.001**	**.208**
CC × CA	1, 77	474.36	<1	.738	.001
Pr × PA	2, 154	544.96	1.54	.218	.020
Pr × PA × CA	2, 154	544.96	<1	.541	.008
Pr × CC	**2, 154**	**692.61**	**37.43**	**<.001**	**.327**
Pr × CC × CA	**2, 154**	**692.61**	**5.31**	**.006**	**.064**
PA × CC	1, 77	499.03	<1	.776	.001
PA × CC × CA	1, 77	499.03	<1	.378	.010
Pr × PA × CC	2, 154	621.33	<1	.673	.005
Pr × PA × CC × CA	**2, 154**	**621.33**	**11.26**	**<.001**	**.128**

Bold indicates significant effect at alpha = .05

**Table 4:** Response times (in millisecond) in Session 2 (values in parentheses are standard errors of the means)

	Compatible	Incompatible
Inducer actor		
After diagnostic actor		
Equal proportion	340 (4.26)	350 (4.61)
Mostly compatible	349 (5.69)	370 (8.15)
Mostly incompatible	372 (7.23)	359 (5.36)
After inducer actor		
Equal proportion	330 (4.34)	339 (5.12)
Mostly compatible	334 (5.66)	374 (8.52)
Mostly incompatible	364 (7.24)	339 (5.39)
Diagnostic actor		
After diagnostic actor		
Equal proportion	328 (4.28)	333 (5.05)
Mostly compatible	338 (5.62)	342 (8.05)
Mostly incompatible	336 (7.14)	339 (5.29)
After inducer actor		
Equal proportion	330 (4.21)	339 (4.56)
Mostly compatible	332 (5.58)	361 (8.42)
Mostly incompatible	355 (7.15)	340 (5.32)

There was a significant Simon effect; RTs were 342 and 349 ms for compatible and incompatible trials, respectively. The Simon effect was largest for the mostly compatible block (24 ms), was intermediate for the equal proportion block (8 ms), and was reversed for the mostly incompatible block (−12 ms). The effect of trial proportion was larger for the inducer actor (Simon effects = 31, 9, and −19 ms) than for the diagnostic actor (Simon effects = 17, 6, and −6 ms). Furthermore, the effect of trial proportion for the two actors depended on the actor performing the preceding trial. To disentangle this interaction, the proportion effect was examined separately for trials following the inducer actor and for trials following the diagnostic actor in terms of 3 (Proportion: mostly compatible vs. equal proportion vs. mostly incompatible) × 2 (Current Compatibility: compatible vs. incompatible) ANOVAs for the two actors.

For the inducer actor (see Fig. 2a), the effect of trial proportions on the Simon effect (i.e., the Proportion × Current Compatibility interaction) was apparent on trials that followed trials in which the inducer actor was active, *F*(2, 76) = 15.73, MSE = 1322.97, *p* < .001, *η*_p_^2^ = .293, and on trials that followed trials in which the diagnostic actor was active, *F*(2, 76) = 8.13, MSE = 720.44, *p* = .001, *η*_p_^2^ = .176. More importantly for our purposes, for the diagnostic actor (see Fig. 2b), the effect of trial proportion on the Simon effect was apparent only on trials that followed trials in which the inducer actor was active, *F*(2, 78) = 28.14, *MSE* = 353.55, *p* < .001, *η*_p_^2^ = .419, but not on trials that followed trials in which the diagnostic actor was active, *F*(2, 78) < 1, MSE = 249.61, *p* = .991, *η*_p_^2^ < .001.

## Discussion

The present study examined to what degrees performing a joint task entails representing the co-actor’s task parameters. Previous studies suggested that the actors represent stimuli and responses of their active co-actor (see Dolk et al., 2014). We asked whether they also represent other aspects of the co-actor’s task-set, such as stimulus–response mappings and frequencies of particular trial events. Our experimental set up in Session 1 was successful in reproducing the standard joint Simon effect: the Simon effect was present in the joint task but not in the individual task (Sebanz et al., 2003). It was also successful in reproducing previously obtained effects of compatibility on the preceding trial in the individual and joint tasks (Liepelt et al., 2011). An important manipulation of the study was the proportion of compatible and incompatible trials for the inducer actor in Session 2. This manipulation allowed for the comparison of performance after trials in which the diagnostic actor performed the task and in which the inducer actor performed it. We consider two observations in Session 2 that are particularly diagnostic in gaining a better understanding of *what* exactly is shared by co-acting individuals.

First, for the diagnostic actor, there was not any sign of an impact of the proportion-of-compatibility manipulation on trials that followed the diagnostic actor’s own performance. This suggests that the diagnostic actor did not keep track of the probabilities of stimuli, responses, and stimulus–response compatibility relations of the inducer actor. As the performance of the inducer actor did depend on the proportion-of-compatibility manipulation regardless of whether the trial followed the inducer actor’s own trial or the diagnostic actor’s, the inducer actor did keep track of these probabilities related to his or her own trials. Consequently, the results imply that the task-sets of the two actors were different and, thus, were not shared. Instead, findings suggest that each actor only kept track of the trial proportions for their own trials and used that information to adjust their contextualized task-sets.

A previous study of the individual task has suggested that the actors can adjust their task-sets based only on the instructions that mention different proportions of compatible and incompatible trials without actually experiencing the different proportions (Entel, Tzelgov, & Bereby-Meyer, 2014). Given such a finding, it is interesting that the actors in our study did not adjust their task-sets even when they observed the different proportions of compatible and incompatible trials for their co-actor.^2^ Thus, to adjust their task-sets, it is important that the actors expect different proportions of their own trials, not those of their co-actor’s trials. These results are inconsistent with a strong claim that actors in a joint task represent their co-actors’ actions as if they were their own (Knoblich & Sebanz, 2006; Knoblich et al., 2011). An alternative view that has recently been proposed to explain the joint Simon effect argues that actors in a joint task represent their own action as reference to their co-actor’s action (e.g., Dittrich et al., 2013; Dolk et al., 2014). This *referential coding account* implies that the actors represent their co-actor’s action but not as their own response but as a reference point of their own action. If so, the actors would not need to monitor the co-actor’s trials and do not share the task-set. The present results are consistent with the account.

Second, the diagnostic actor’s performance was affected by the proportion-of-compatibility manipulation if the diagnostic actor was performing right after a trial of the inducer actor. This outcome does not reflect any knowledge of the diagnostic actor regarding the inducer actor’s task-set, as indicated by the lack of the proportion-of-compatibility manipulation on trials that followed the diagnostic actor’s own trials. Instead, the outcome reflected the greater frequency of compatible or incompatible trials that directly preceded the diagnostic actor’s own action, which was shown in Session 1. As we have mentioned in the “Introduction”, there are two possible reasons for previous compatibility to modulate the Simon effect, which are not necessarily mutually exclusive. According to the first account, the effect of previous compatibility represents a readjustment of task-set parameters (e.g., Botvinick et al., 2004): performing an incompatible trial creates cognitive conflict that is reduced or resolved by increasing the focus on relevant information. In the Simon task, this would lead to a reduction of the relative impact of the irrelevant stimulus location, which would reduce the Simon effect on the next trial. According to this interpretation, the present results would indicate that control adjustments are stronger or more likely to occur after a trial for which the actor did not perform the task (nogo trial or co-actor’s trial). This is possible, as it might be that inhibiting one’s own action creates cognitive conflict that leads to a stronger adjustment than if one was performing the task by oneself (cf. Sebanz, Knoblich, Prinz, & Wascher, 2006). According to the second account, the effect of previous compatibility reflects the automatic retrieval of feature bindings created in the previous trial (Hommel et al., 2004). It has been shown that engaging in a trial leads to the integration of stimulus and response features into event files, and these event files are retrieved automatically when these features repeat on the next trial (Hommel, 2004). Partial repetitions are particularly problematic, whereby only one of these features repeats but the other alternates, which results in retrieval of two conflicting event files. Stimulus and response repetitions or alternations are confounded with the combination of previous compatibility and present compatibility that is relevant for the control-adjustment account (Hommel et al., 2004).

The effect of previous compatibility in Session 1 may actually reflect the automatic creation and retrieval of event files. Consider a setup in which the actor is sitting on the left and engages in a go/nogo task. We have seen that such a situation leads to particularly elevated RTs after a non-active trial if the current trial is compatible and the previous trial was incompatible, or if the current trial is incompatible and the previous trial was compatible. If the previous trial was incompatible, this means that a stimulus on the left appeared and signaled a right-hand response, which was then either not executed (in the individual task) or executed by the co-actor (in the joint task). The actor could thus be assumed to store this event by creating a binding between LEFT STIMULUS and either RIGHT RESPONSE, OTHER ACTOR, NOT RESPONDING, or any mixture of those. If then a left stimulus would indicate a left-hand response on the next trial, the repetition of the LEFT STIMULUS feature would tend to retrieve the previously associated features (i.e., those coding for the right response, the other actor, not responding, or all of that), which in any case would conflict with the selection of the correct left response that the current actor is expected to perform. A similar scenario applies to an incompatible trial that follows a passive compatible trial: a stimulus presented on the right and indicating a right-hand response would induce a binding between RIGHT STIMULUS and either RIGHT RESPONSE, OTHER ACTOR, NOT RESPONDING, or any mixture of those. Repeating the stimulus location, as in an incompatible trial following that, would increase response conflict for the same reasons. Although our study was not designed to disentangle the two accounts, we believe that the present outcome pattern provides theoretically interesting constraints for our understanding of the effect.

We thus conclude that people are directly affected by the stimuli that are relevant for their co-actor and by the actions that their co-actor performs, but they do not seem to be sensitive to a number of rather crucial ingredients of their co-actor’s task-sets, namely, stimulus and response probabilities and the probability of the resulting stimulus–response compatibility relations. While we admit that the chosen experimental design was rather complex, as were some of the considerations necessary to disentangle theoretically relevant factors and effects, we do see the present study as a encouraging step towards a better understanding of whether and to what degree people represent their co-actor’s task parameters (also see Yamaguchi et al., 2016). The present study does not require the assumption that the degree of sharing goes anywhere beyond currently available (and not dedicatedly social) information, but task-sets can contain other information than stimulus, response, and stimulus–response probabilities. The present results suggest that the co-acting individuals are only concerned about their part of the joint Simon task but not much about their co-actor’s part. This calls for more functionally analytic studies addressing the possibility of task-set sharing.

## Footnotes

1 We have also conducted a separate ANOVA that included the order of blocks (Order: mostly compatible first vs. mostly incompatible first; between-subject) in addition to those factors in the main analysis. There were three significant interactions that involved Order. First, Order interacted with Proportion, *F*(2, 150) = 3.53, *p* = .032. For the Mostly Comp First group RTs were 334, 341, and 345 ms, for the equal proportion, mostly compatible, and mostly incompatible blocks, respectively. For the Mostly Incomp First group, RTs were 338, 360, and 357 ms, for the equal proportion, mostly compatible, and mostly incompatible blocks, respectively. Thus, between the two biased proportion blocks, RT tended to be faster (by 3–4 ms) in the first block than in the second block. Second, there was a 3-way interaction among Proportion, Previous Actor, and Order, *F*(2, 150) = 4.38, *p* = .014, and a 4-way interaction among Proportion, Previous Actor, Order, and Current Actor, *F*(2, 150) = 3.20, *p* = .043. The 3-way interaction reflected the fact that the advantage of the block that was performed first disappeared when the same actor repeated. The 4-way interaction reflected the fact that the advantage of the block that was performed first was obtained only for the inducer actor but not for the diagnostic actor. These outcomes indicate that the inducer actor reacted to the trial proportions, but the diagnostic actor was not sensitive to the trial proportions for the inducer actor, supporting our conclusion based on the main analysis.

2 We thank Kerstin Dittrich for pointing this out.

## References

[CR1] Bedwell WL, Wildman JL, DiazGranados D, Salazar M, Kramer WS, Salas E (2012). Collaboration at work: an integrative multilevel conceptualization. Human Resource Management Review.

[CR2] Botvinick MM, Cohen JD, Carter CS (2004). Conflict monitoring and anterior cingulate cortex: an update. Trends in Cognitive Sciences.

[CR3] Dittrich K, Dolk T, Rothe-Wulf A, Klauer KC, Prinz W (2013). Keys and seats: spatial response coding underlying the joint spatial compatibility effect. Attention, Perception, & Psychophysics.

[CR4] Dolk T, Hommal B, Colzato LS, Schütz-Bosbach S, Prinz W, Liepelt R (2014). The joint Simon effect: A review and theoretical integration. Frontiers in Psychology.

[CR100] Entel O, Tzelgov J, Bereby-Meyer Y (2014). Proportion congruency effects: Instructions may be enough. Frontiers in Psychology.

[CR5] Hommel B (1994). Spontaneous decay of response code activation. Psychological Research.

[CR6] Hommel B (1996). S-R compatibility effects without response uncertainty. Quarterly Journal of Experimental Psychology.

[CR7] Hommel B (2004). Event files: feature binding in and across perception and action. Trends in Cognitive Sciences.

[CR8] Hommel B (2011). The Simon effect as tool and heuristic. Acta Psychologica.

[CR9] Hommel B, Proctor RW, Vu K-PL (2004). A feature integration account of sequential effects in the Simon task. Psychological Research.

[CR10] Knoblich G, Butterfill S, Sebanz N, Ross B (2011). Psychological research on joint action: Theory and data. The psychology of learning and motivation.

[CR11] Knoblich G, Sebanz N (2006). The social nature of perception and action. Current Direction in Psychological Science.

[CR12] Liepelt R, Wenke D, Fischer R (2013). Effects of feature integration in a hands-crossed version of the Social Simon paradigm. Psychological Research.

[CR13] Liepelt R, Wenke D, Fischer R, Prinz W (2011). Trial-to-trial sequential dependencies in a social and non-social Simon task. Psychological Research.

[CR14] Lu C-H, Proctor RW (1995). The influence of irrelevant location information on performance: a review of the Simon and spatial Stroop effects. Psychonomic Bulletin & Review.

[CR15] Proctor RW, Yamaguchi M, Dutt V, Gonzalez C (2013). Dissociation of S-R compatibility and Simon effects with mixed tasks and mappings. Journal of Experimental Psychology: Human Perception and Performance.

[CR16] Sebanz N, Knoblich G, Prinz W (2003). Representing others’ actions: Just like one’s own?. Cognition.

[CR101] Sebanz N, Knoblich G, Prinz W (2005). How two share a task: Corepresenting stimulus-response mappings. Journal of Experimental Psychology: Human Perception and Performance.

[CR17] Sebanz N, Knoblich G, Prinz W, Wascher E (2006). Twin peaks: An ERP study of action planning and control in coacting individuals. Journal of Cognitive Neuroscience.

[CR18] Stürmer B, Leuthold H, Soetens E, Schröter H, Sommer W (2002). Control over location-based priming in the Simon task: Behavioral and electrophysiological evidence. Journal of Experimental Psychology: Human Perception and Performance.

[CR19] Wühr P, Duthoo W, Notebaert W (2015). Generalizing attentional control across dimensions and tasks: Evidence from transfer of proportion-congruent effects. Quarterly Journal of Experimental Psychology.

[CR20] Yamaguchi M, Proctor RW (2012). Multidimensional vector model of stimulus-response compatibility. Psychological Review.

[CR21] Yamaguchi M, Wall HJ, Hommel B (2016). No evidence for shared representations of task sets in joint task switching. Psychological Research.

